# A genomic-clinicopathologic Nomogram for the preoperative prediction of lymph node metastasis in gastric cancer

**DOI:** 10.1186/s12885-021-08203-x

**Published:** 2021-04-23

**Authors:** Xin Zhong, Feichao Xuan, Yun Qian, Junhai Pan, Suihan Wang, Wenchao Chen, Tianyu Lin, Hepan Zhu, Xianfa Wang, Guanyu Wang

**Affiliations:** Department of General Surgery, Sir Run Run Shaw Hospital, School of Medicine, Zhejiang University, East Qingchun Road 3, Zhejiang, 310016 Hangzhou China

**Keywords:** Gastric cancer, Gene signature, Nomogram, Lymph node metastasis

## Abstract

**Background:**

Preoperative evaluation of lymph node (LN) state is of pivotal significance for informing therapeutic decisions in gastric cancer (GC) patients. However, there are no non-invasive methods that can be used to preoperatively identify such status. We aimed at developing a genomic biosignature based model to predict the possibility of LN metastasis in GC patients.

**Methods:**

We used the RNA profile retrieving strategy and performed RNA expression profiling in a large GC cohort (GSE62254, *n* = 300) from Gene Expression Ominus (GEO). In the exploratory stage, 300 GC patients from GSE62254 were involved and the differentially expressed RNAs (DERs) for LN-status were determined using the R software. GC samples in GSE62254 were randomly allocated into a learning set (*n* = 210) and a verification set (*n* = 90). By using the Least absolute shrinkage and selection operator (LASSO) regression approach, a set of 23-RNA signatures were established and the signature based nomogram was subsequently built for distinguishing LN condition. The diagnostic efficiency, as well as the clinical performance of this model were assessed using the decision curve analysis (DCA). Metascape was used for bioinformatic analysis of the DERs.

**Results:**

Based on the genomic signature, we established a nomogram that robustly distinguished LN status in the learning (AUC = 0.916, 95% CI 0.833–0.999) and verification sets (AUC = 0.775, 95% CI 0.647–0.903). DCA demonstrated the clinical value of this nomogram. Functional enrichment analysis of the DERs was performed using bioinformatics methods which revealed that these DERs were involved in several lymphangiogenesis-correlated cascades.

**Conclusions:**

In this study, we present a genomic signature based nomogram that integrates the 23-RNA biosignature based scores and Lauren classification. This model can be utilized to estimate the probability of LN metastasis with good performance in GC. The functional analysis of the DERs reveals the prospective biogenesis of LN metastasis in GC.

**Supplementary Information:**

The online version contains supplementary material available at 10.1186/s12885-021-08203-x.

## Background

Globally, gastric cancer (GC) is the 5th most prevalent cancer type and the 3rd highest cause of cancer-associated mortalities [1]. Some studies demonstrated that Lymph node (LN) metastasis is an independent risk index for poor prognosis of GC [2, 3]. Precise and exact preoperative identification of LN involvement is important in informing therapeutic decisions for GC patients [4, 5]. Clinicopathologic factors such as lymphatic invasion or pathological differentiation are associated with LN metastasis, however, they can hardly be obtained preoperatively [6, 7]. The current preoperative prediction of LN metastasis primarily relies on morphological features of the lymph nodes as revealed by computed tomography (CT), which has unfavorable sensitivity [8]. Tumor biosignatures, including carcinoembryonic antigen (CEA), as well as carbohydrate antigen 199 (CA-199) have been shown to be poor predictors of LN metastasis in GC [9, 10]. Therefore, novel diagnostic biomarkers are needed to improve on the current strategies for predicting LN metastasis in GC patients. Gene expression studies have been performed to elucidate on the distinct molecular biosignatures for LN metastases. Daisuke Izumi et al. proposed a 15-gene signature for identification LN metastasis in GC [9]. Song et al. developed a co-expression network of RNAs for assessing LN metastasis in GC patients [11]. These studies show that genes have a high predictive power for detecting LN metastasis. However, clinicopathologic factors associated with LN status were not involved in these studies [12–14]. A Nomogram is a visual predictive tool used to quantify risk factors of LN metastasis in several carcinomas [15, 16], including early GC [17]. However, the current nomogram only integrates clinical and postsurgical factors, which would restrict their clinical value. Therefore, we aimed to establish and verify the efficacy of a nomogram that integrates both gene biosignatures and clinicopathologic parameters for the preoperative prediction of LN metastasis in GC.

## Methods

### Data preparation and differential expression analysis

**Fig. 1:**
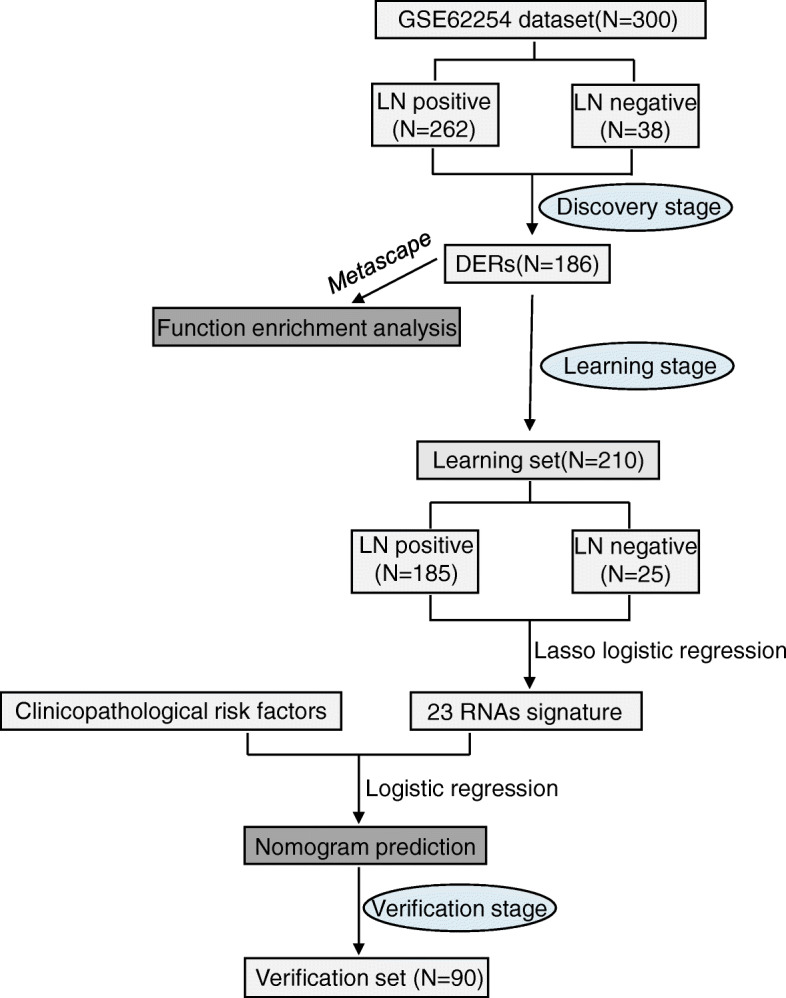
The main flowchart of this study. The flowchart of analyses to establish the nomogram model and test its predictive value. Abbreviations: LN: lymph node, DERs: differentially expressed RNAs,LASSO: Least absolute shrinkage and selection operator

Gene expression information and sample data from GSE62254 dataset in this research were retrieved from GEO (http://www.ncbi.nlm.nih.gov/geo/)in its processed format, using the package ‘GEOquery’ in R. The overview of the screening strategy used in this study is shown in Fig. 1. The clinical data for these samples were downloaded from the authors’ website (https://www.nature.com/articles/nm.3850) on May 20th, 2020. The dataset obtained from the GEO database had been anonymized and, therefore, ethical approval was waived. The samples in GSE62254 were randomly clustered into a learning set and a verification set.

Human gene annotation files (GRCh38.p12) were obtained from the Ensembl repository (https://asia.ensembl.org/index.html) for RNAs annotation on May 20th, 2020. Samples in the GSE62254 dataset were divided into LN-negative and LN-positive arms according to the source information. The differentially expressed RNAs (DERs) were identified using the package limma [18]. DERs were distinguished between the two groups according to the false discovery rate (FDR) < 0.05. Based on the R package heatmap, hierarchical clustering analysis was performed [19]. A volcano plot was developed by the ggplot2 package [19].

### Development of the 23-RNA signature

The least absolute shrinkage and selection operator (LASSO) regression approach which is applicable in the regression analysis of high-dimensional data was performed using the R package “glmnet” [20]. For high-dimensional data with few true predictors and many noise variables, LASSO shrinkage penalty would force a feature weight to zero and this could reduce variables. This is an advantage over ridge regression, as it greatly improves model interpretability [20]. According to the optimal lambda value acquired using cv.glmnet, candidate genes with corresponding coefficients (β_i_) were screened out from the DERs. For each gene, univariate analysis was performed to investigate the association between gene expression levels and lymph node metastasis levels. A risk score was calculated for each patient using the linear combination of expression data (Exp_i_) of selected genes that were weighted by their corresponding coefficients (β_i_) and intercept. Based on the above process, a risk-score formula was developed as:

Risk score (RS) = $\sum \limits_{\mathrm{i}=1}^{\mathrm{n}=23}$ (β_i_ × Exp_i_) + Intercept

The R package “OptimalCutpoints” was applied in determining the optimum cutoff point for risk score. The optimum cutoff was employed to cluster the patients into high- or low-risk classes. It was obtained when the Youden index in receiver operating characteristic (ROC) curve predicting LN metastasis reached its maximum in the learning set. Samples were clustered into high- or low-risk clusters by utilizing the optimum cutoff.

### Construction and assessment of genomic signature based model

Candidate predictors including age, sex, Borrmann classification, Lauren classification, tumor location and the risk score were embedded into the logistic regression analysis to design a diagnostic model for predicting LN metastasis in the learning set [21, 22]. To provide a quantitative technique for predicting individual likelihood of LN metastasis, a nomogram prediction model was constructed based on the independent risk factors using the R package rms [23]. Receiver operating characteristic (ROC) assessment was performed to inspect the sensitivity and specificity of the nomogram using R package “pROC” [24]. The calibration curve was subsequently utilized to examine the effectiveness of the nomogram with additional 1000 bootstrap samples to decrease the over fit bias. Decision curve analysis (DCA) was applied to inspect the clinical application of the gene signature based model [25].

### Functional enrichment analysis

Metascape (http://metascape.org/gp/index.html) was used to predict the potential biological functions of the differentially expressed genes [26].

### Statistical analyses

A chi-square test was used for the analysis of categorical variables between the two sets. The Student’s t test was applied in continuous variables assessments. Statistical analyses were performed using the SPSS software (version 24) or R software (version3.5.3). All tests were dual-sided and *P*-value below 0.05 signified statistical significance.

## Results

### Patient characteristics

Samples in the GSE62254 dataset were randomly clustered into a learning set (*n* = 210, Additional file 1) and a verification set (*n* = 90, Additional file 2). The baseline features of all patients are shown in Table 1. The LN metastasis incidences were 88.1% in the learning set and 85.6% in the verification sets with no significant differences.

**Table 1:** Baseline features of all subjects

	Learning set(n = 210)	Verification set(n = 90)	pValue
Sex			0.539754
Male	137	62	
Female	73	28	
Age			0.667548
> =65	111	50	
< 65	99	40	
Borrmann			0.560256
B-I	11	5	
B-II	70	34	
B-III	98	43	
B-IV	31	8	
Lauren			0.216635
Intestinal	91	51	
Mixed	13	4	
Diffuse	101	35	
Location			0.787408
Antrum	108	47	
Body	73	34	
Cardia	24	8	
Whole	5	1	
N			0.544463
Negative	25	13	
Positive	185	77	

Categorical variables were compared by Chi squared test or Fisher’s exact test as appropriate

LN, lymph node;

### Differential expression analysis

**Fig. 2:**
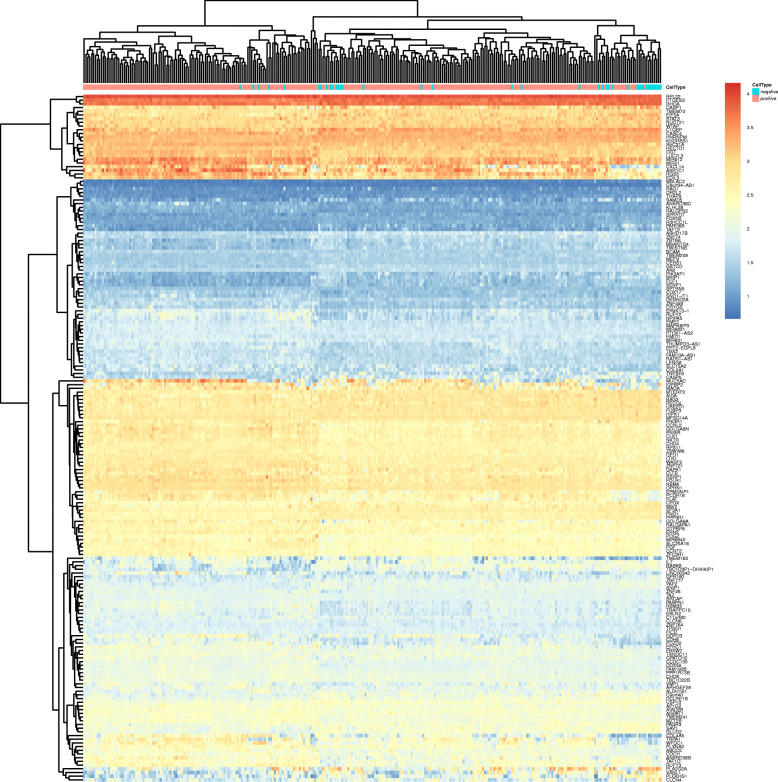
Heatmap: The hierarchical clustering heatmap (pink and blue represent lymph node positive and the lymph node negative samples, respectively in sample strip)

**Fig. 3:**
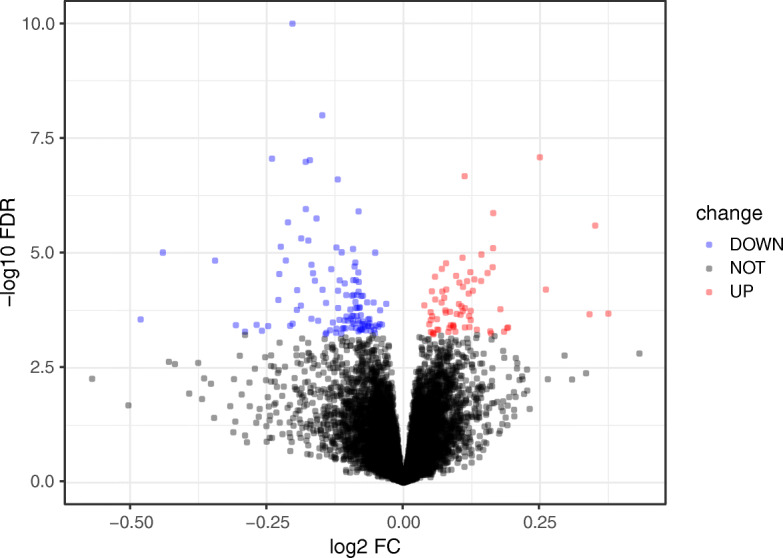
Volcano plot: The volcano plot (the red and blue dots represent up- and down-regulation of differentially expressed RNAs respectively, false discovery rate < 0.05)

Overall, 14,651 mRNAs, 840 lncRNAs, and 111 miRNAs were annotated from the GSE62254 datasets. The 300 GC samples in the GSE62254 dataset were allocated into LN-negative (38 samples) and LN-positive (262 samples) groups. 186 DERs (Additional file 3) were screened out under the defined thresholds between the LN-positive and the LN-negative groups. Among the 186 DERs, 70 DERs were found to be upregulated while 116 DERs were downregulated. Based on expression of the DERs, the heatmap and volcano plot are shown, in Fig. 2 and Fig. 3, respectively.

### Construction of 23-RNA signature based risk score

**Fig. 4:**
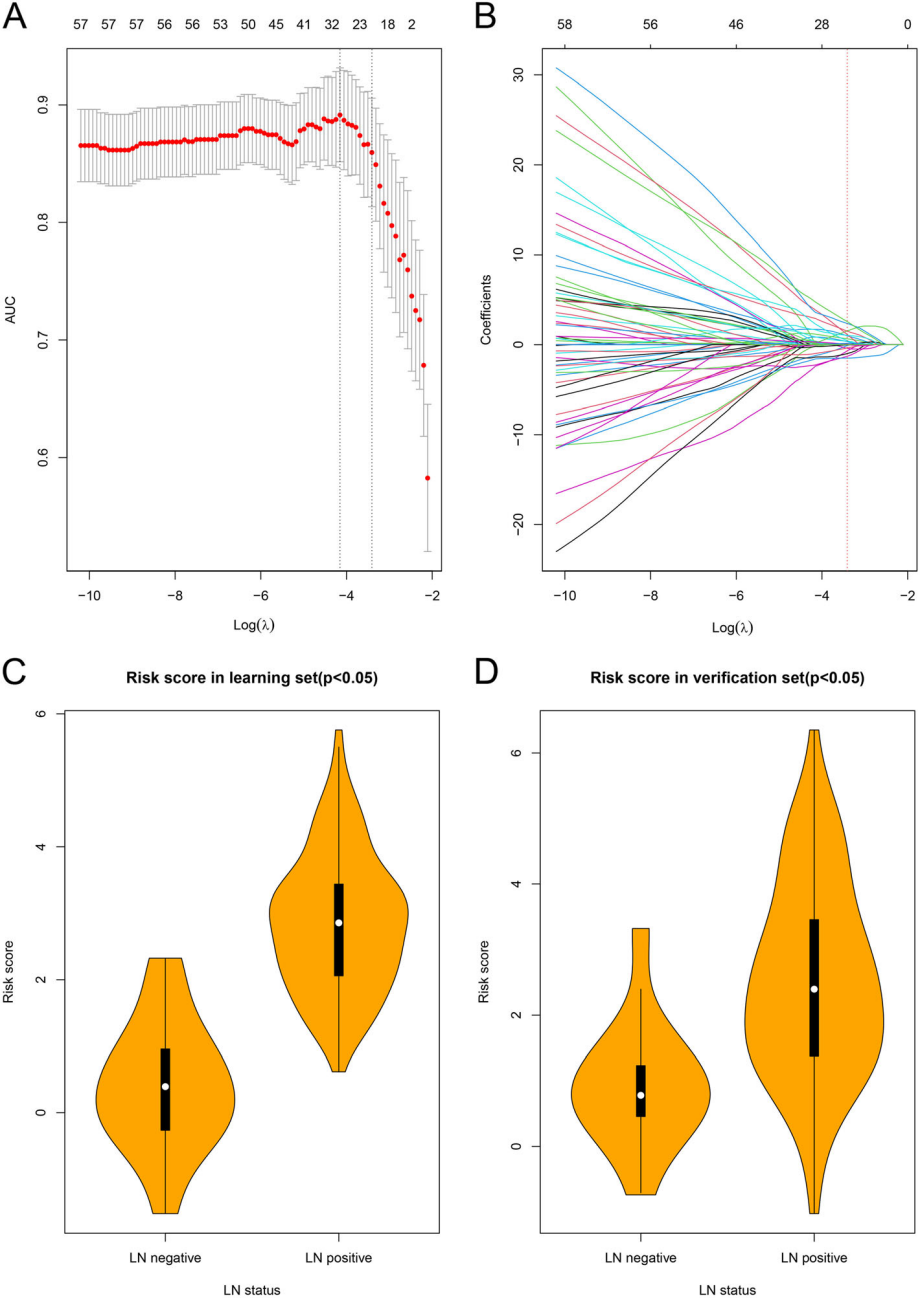
Selection of the genes trough the LASSO approach and distribution of risk score. (a) Selection of tuning parameter (λ) via 10-fold cross-verification with minimum criteria. The area under curve was plotted versus log (λ). Dotted vertical lines were drawn at the optimal values using the minimum criteria and the 1 standard error of the minimum criteria (the 1-SE criteria). The optimal λ value of 0.033, with log (λ) = − 3.411 was chosen based on minimum criteria. (b) LASSO coefficient profiles of the 186 differentially expressed RNAs. A coefficient profile plot was generated against the log (λ) sequence. Vertical line was drawn at the optimal value where optimal λ led to 23 nonzero coefficients. (c) Distribution of risk score in learning set. (d) Distribution of risk score in verification set

A total of 186 DERs with non-zero coefficients in the LASSO logistic regression model were reduced to 23 RNAs on the basis of 210 patients in the learning set (Additional file 5) (Fig. 4a, b). The risk score formula was subsequently established based on the 23 RNAs and their corresponding coefficients (Additional file 4 / Table 1s). The developed formula is:

RS = 0.3370*ExpTRAPPC10 + (− 0.6895)*ExpRHOA + 0.0452*ExpIGFBP2 + 1.4984*ExpC11orf80 + (− 0.0937)*ExpZNF74 + (− 0.9888)*ExpFOXN2 + 0.6580*ExpGOLGA8A + 0.9803*ExpRSRP1 + (− 0.4094)*ExpUSP10 + 0.3896*ExpCLTB + (− 1.2924)*ExpPIK3R1 + 1.5335*ExpPABPN1 + (− 0.3669)*ExpCLCN4 + (− 1.4978)*ExpPARD6B + 0.0329*ExpTRPA1 + (− 0.0174)*ExpBAG3 + 0.4511*ExpZNF26 + 0.0381*ExpGDPD3 + 1.1286*ExpSPTBN5 + 2.3647*ExpKLHL28 + 1.0420*ExpGTPBP8 + 2.5667*ExpTXNDC11 + 0.1489*ExpTMEM163 + Intercept.

We also compared the expression of each of the 23 genes between LN-positive and the LN-negative groups. Most of the genes were correlated with LN metastasis (*p* < 0.05 Additional file 4/ Table 1s).

The distribution of risk scores between LN-negative and LN-positive groups with significant differences (*p* < 0.05) are shown, in Fig. 4c and d, respectively. The cutoff value of the risk scores was calculated, and the samples were separately clustered into high or low risk classes in both the learning and verification sets. The cutoff value (1.3806) was obtained when the ROC curve reached optimum sensitivity (94.05%) and specificity (88.00%) for predicting LN metastasis (Additional file 5/ Fig. S1a). The Positive Predictive Value (PPV) reached 98% (Additional file 5/ Fig. S1b). Patients in the learning set with a risk score higher than 1.3806 were assigned to the high-risk group (*n* = 177) while the rest (*n* = 33) were assigned to the low-risk group (Additional file 6). Patients in the verification set with a risk score higher than 1.3806 were assigned to the high-risk group (*n* = 60) while the rest (*n* = 30) were assigned to the low-risk group (Additional file 7).

### Construction and verification of genomic signature based model

**Fig. 5:**
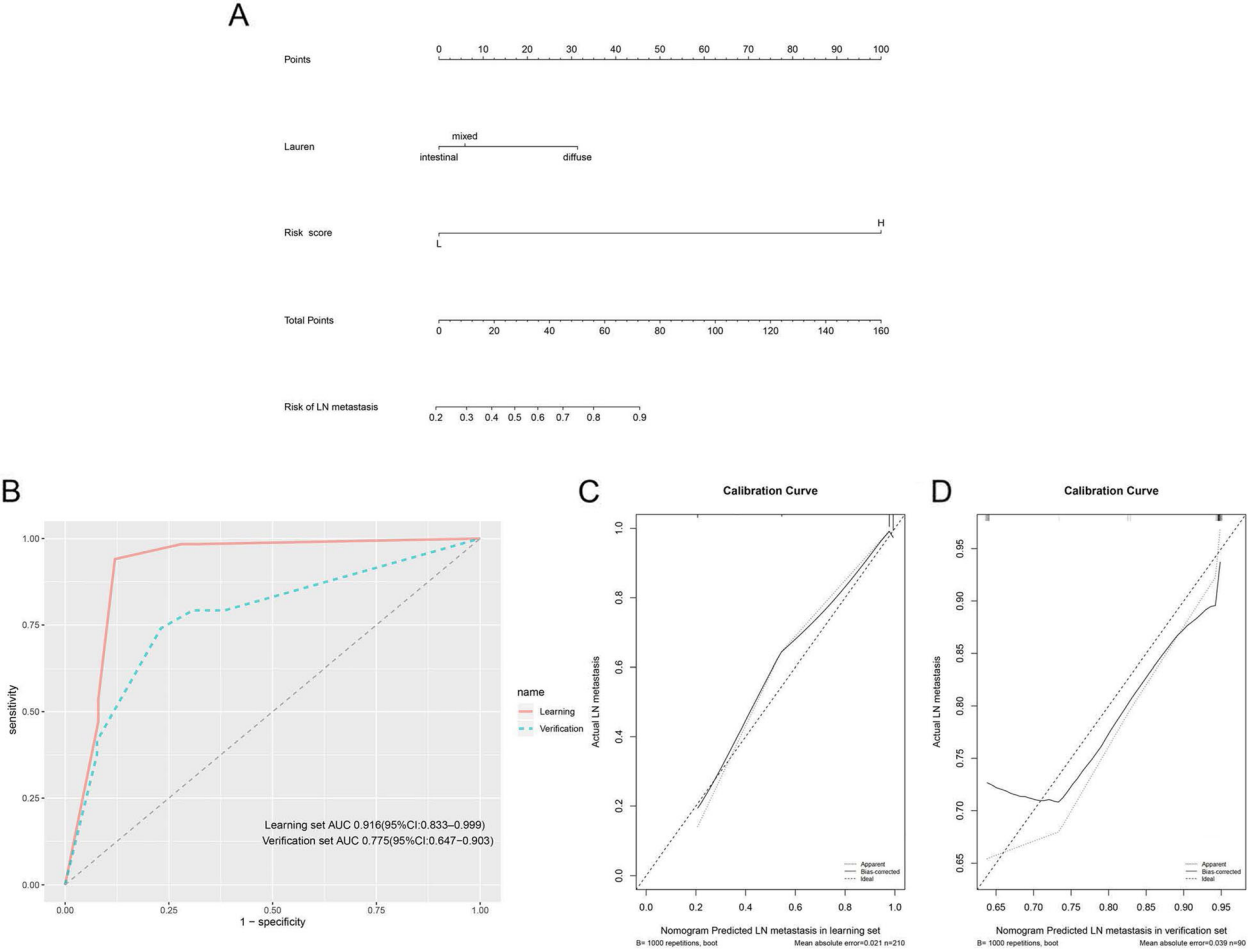
Developed a genomic signature based nomogram and the performance of the nomogram. (a) The nomogram was designed in the learning set, with the 23-mRNA biosignature based risk score and Lauren classification integrated. (b) The area under curve of nomogram in learning set was 0.916 (95% CI: 0.833–0.999). Area under curve of nomogram in verification set was 0.775 (95% CI: 0.647–0.903). (c) Calibration plot in learning set (mean absolute error = 0.021). After 1000 repetitions of bootstrap, the bias-corrected calibration curve (solid line) was close to the ideal curve (dashed line). (d) Calibration plot in verification set (mean absolute error = 0.039)

By using the logistic regression analysis, Lauren classification (odds ratio [OR] = 2.126, 95% CI 1.070–4.223, p < 0.05) and risk score (OR = 126.126, 95%CI 30.466–522.148, p < 0.05) were confirmed as independent risk factors for LN metastasis (Table 2). Based on the two independent predictive factors, a nomogram model was subsequently built (Fig. 5a). LN metastasis probability was easily calculated based on their Lauren classification and risk scores. ROC evaluation was used to examine sensitivity and specificity of the nomogram. It was found that the nomogram had an optimum sensitivity of 94.1% and specificity of 88.0% when predicting LN metastasis in the learning set, and an optimum sensitivity of 74% and specificity of 76.9% in the verification set. The area under curve (AUC) were 0.916 (95% CI: 0.833–0.999) for learning set and 0.775 (95% CI: 0.647–0.903) for the verification set, which implied that the nomogram had good utility (Fig. 5b). In addition, the predicted probability of LN metastasis was further compared with the authentic probability by the calibration curve in the learning and verification set. Deviation when probability was below 75% in the verification group, bias-corrected calibration plot of the nomogram corresponded closely with the authentic probability in both sets. These findings of the estimated likelihood of LN metastasis and authentic probability were consistent. The mean absolute errors were 0.021 and 0.039 in the learning and verification set respectively (Fig. 5c, d). The DCA for genomic-clinicopathologic nomogram demonstrated that if the threshold ranged from 0.20 to 0.95, the nomogram model was more beneficial relative to either the treat-all-cases scheme or the treat-none scheme (Additional file 8/Fig. S2).

**Table 2:** Multivariate evaluations to evaluate potential predictive factors for LN metastasis

	Univariable p	Multivariable p	OR	95% CI
Sex	0.250			
Age	0.866			
Borrmann	0.729			
Lauren	0.054	0.031	2.126	1.070–4.223
Location	0.894			
Risk score	< 0.0001	< 0.0001	126.126	30.466–522.148

LN, lymph node; OR, odds ratio; CI, confidence interval

### Functional enrichment analysis

**Fig. 6:**
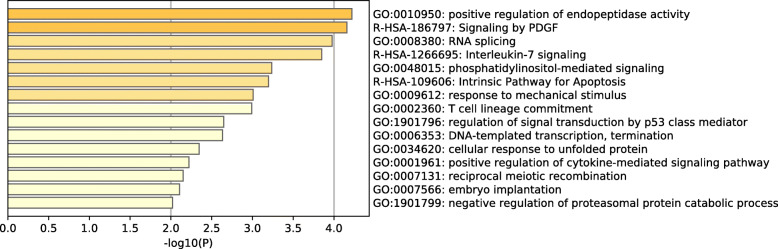
Bar graph of enriched terms across the differentially expressed RNAs, colored by *p*-values

Metascape was used for cascade and process enrichment analysis of the DERs (Additional file 9). The top 15 clusters with their illustrative enriched terms are shown in Fig. 6. A sub-cluster of the enriched terms was selected and regarded as a network plot (Additional file 10/Fig. S3). Specifically, the enriched DERs were associated with several pathways, such as Signaling by platelet derived growth factor (PDGF) and Intrinsic Pathway for Apoptosis.

## Discussion

LN metastasis is involved in GC prognostic outcomes [2, 3]. Precise preoperative determination of LN involvement in GC is pivotal for clinical decision-making. Less invasive therapeutic options such as endoscopic submucosal resection (ESD) can be effectively performed for LN negative patients in early GC. However, ESD should be avoided for early GC patients with a high risk of LN metastasis [27, 28]. For localized LN negative GC patients, limited LN resection is recommended to reduce postoperative complications. Surgical resection with extensive lymphadenectomy is necessary for advanced GC patients with LN metastasis [5]. Therefore, it is important to accurately determine the extent and degree of LN metastasis in order to inform therapeutic decisions. With the development of high throughput sequencing (HTS) technologies, the molecular portrait of GC has been comprehensively analyzed by gene-expression profiling [29, 30]. As RNA-sequencing technology provides molecular insights into tumor biology process, we focused on building a genomic signature based Nomogram for predicting LN metastasis in GC. By using cDNA microarrays, several studies have reported certain geneexpression-based biomarkers for predicting LN metastasis in GC [31–33]. However, these studies did not elucidate on the clinical characteristics associated with LN status in GC [12–14].

Based on the Lauren classification, GC can be grouped into intestinal or diffuse kinds [34]. The intestinal type of GC stems from premalignant lesions developed from an initial *Helicobacter pylori* (*H. pylori*) triggered chronic gastritis and successive atrophic and metaplastic gastritis [35]. The diffuse form of GC is triggered by active inflammation of the gastric mucosa [36, 37]. Diffuse forms are prevalent in younger patients with an elevated risk of LN metastasis compared to the intestinal types [38–40]. Our study established that Lauren classification was an independent risk index for LN metastasis while diffuse type was associated with elevated risk of LN metastasis relative to the intestinal form.

We constructed and verified a diagnostic, genomic biosignature based nomogram as a noninvasive strategy for preoperative estimation of LN metastasis in GC. This nomogram incorporates two items of genomic signature based risk scores and Lauren classification. Though deviation was obviously found in the verification set when probability was below 75%, the nomogram exhibited ideal coincidence to the authentic probability in the learning set. The possible reason for deviation observed in the verification set may be the predictive model has an over-fitting problem as it was built based on data from the learning set. Therefore, it did not perform as well in the verification set as it did in the learning set when predicting LN metastasis. The areas under the ROC curve for the learning and verification sets implied that the nomogram had good utility. The DCA is a simple method for evaluating the clinical performance of a prediction model. It can quantify different strategies and determine an optimal threshold range. This LN metastasis prediction model can assist surgeons to balance between the quality of life and aggressive lymphadenectomy.

To provide insights into the potential biological processes, “metascape” was performed for the functional and enrichment analysis of DERs. The DERs were enriched in three signaling pathways, including PDGF signaling, Interleukin-7 signaling and in the Intrinsic pathway for apoptosis. The PDGF receptor cascade constitutes a signaling network that is essential for the growth of cells of mesenchymal parentage. Dysregulation of this pathway can lead to extracellular matrix reconstruction in a tumor-enhancement manner to promote the migration, infiltration, angiogenesis, and lymphangiogenesis [41, 42]. For this pathway, enriched genes such as STAT3 can activate cancer after the interaction of cytokines and cell surface receptors, and regulation of the downstream and promote the proliferation and growth of gene expression [43]. PLAT stimulates plasminogen activator which degrades the extracellular matrix, especially the collagen fiber components, mediating cell migration and tissue remodeling [44]. As for the Interleukin-7 signaling pathway, the Interleukin-7 (IL-7) gene is involved in both B-cell and T-cell proliferation and its absence leads to immature immune cell arrest. IL-7 modulates cell growth, apoptosis and modulates cancer lymphangiogenesis [45, 46]. RAG1 encodes the RAG1 protein which is involved in adjusting the starting phase of V(D) J recombination, making the rearrangement of antigen receptor gene strictly in line with the tissue and cell growth phases [47]. Low RAG1 gene expression is correlated with poor survival of gastric cancer patients [47]. Apoptosis is a form of programmed cell death. Insufficient apoptosis is associated with neoplastic diseases [48]. In the Intrinsic Pathway for Apoptosis, enriched genes such as complement C1q binding protein (C1QBP), also referred to as p32, are expressed in various cancer types [49–54]. Protein phosphatase 3 regulatory factor subunit 1 (PPP3R1) is a member of β-regulatory subunit family of calcineurin that codes for apoptosis-stimulating protein of p53 (ASPP) in the p53 integrin family [55]. The ASPP enhances P53-mediated apoptosis by binding to the P53 core domain [56]. However, the specific molecular mechanisms of the differentially expressed genes in the pathways have not been established. Elucidation of these mechanisms can provide new clues and molecular targets for the identification and specific treatment of GC with LN metastasis.

Compared to previous nomograms [15–17], our model incorporates Lauren classification and genomic signature based risk scores. This model exhibited a high accuracy for predicting LN metastasis. However, there were some limitations associated with this study. First, we did not perform external verification using data from another institution for this model. Second, clinicopathological factors, such as CEA level and CT-reported LN status, were not available in the GSE62254 dataset. Therefore, these important clinical features, could not be examined in this study. More, studies should be performed to elucidate on the functions of DERs in the pathogenesis of LN metastasis.

## Conclusions

In conclusion, this nomogram incorporates both genomic signature based risk score and Lauren classification to estimate LN metastasis in preoperative GC.

## Supplementary Information

**Additional file 1.** Learning set.

**Additional file 2.** Verification set.

**Additional file 3.** 186 DERs were reduced to 23 RNAs on the basis of 210 patients in the learning set.

**Additional file 4 **Table S1 These 23 RNAs with their corresponding coefficients and univariate analysis result between gene expression level and lymph node metastasis level. *p*-values are based on t-test.

**Additional file 5 **Figure S1 The optimum cutoff point of risk score. (**a**) The cutoff value (1.3806) was acquired when the ROC curve reached optimum sensitivity (94.05%) and specificity (88.00%) for predicting LN metastasis. (**b**) The cutoff value (1.3806) was acquired when Positive Predictive Value (PPV) reached 98%.

**Additional file 6.** Risk score and risk status for each sample in Learning set.

**Additional file 7.** Risk score and risk status for each sample in Verification set.

**Additional file 8.** Figure S2 Decision curve analysis for the genomic signature based nomogram.

**Additional file 9.** Metascape Analysis result.

**Additional file 10 **Fig S3 Network of enriched terms: (**a**) Colored by the cluster-ID, in which the nodes with similar cluster ID are frequently close to each other. (**b**) Colored by p-value, in which the terms with more genes tend to have a more remarkable p-value.

## Data Availability

The datasets generated and analysed during the current study are available in the Gene Expression Ominus (GEO) (https://www.ncbi.nlm.nih.gov/geo/query/acc.cgi?acc=GSE62254).

## Abbreviations

GC: Gastric cancer
LN: Lymph node
GEO: the Gene Expression Omnibus
DERs: Differentially expressed RNAs
LASSO: Least absolute shrinkage and selection operator
DCA: Decision curve analysis
AUC: The area under curve.
CT: Computed tomography
ROC: Receiver operating characteristic
FDR: False discovery rate
OR: Odds ratio
CI: Confidence interval
PDGF: Platelet derived growth factor
ESD: Endoscopic submucosal resection
IL-7: Interleukin-7
