# Supplementary material for: A genomic-clinicopathologic Nomogram for the preoperative prediction of lymph node metastasis in gastric cancer
Source: BMC Cancer. 2021 Apr 23;21:455. doi: 10.1186/s12885-021-08203-x (PMC8066490; doi:10.1186/s12885-021-08203-x)
Supplement: Supplementary file 4 — Additional file 4 Table S1 These 23 RNAs with their corresponding coefficients and univariate analysis result between gene expression level and lymph node metastasis level. p-values are based on t-test. [file 12885_2021_8203_MOESM4_ESM.doc]

**Table s1** **23 RNAs with their corresponding coefficients and univariate analysis result**

|  | **coef** | **pvalue** |
| --- | --- | --- |
| TRAPPC10 | 0.337 | 0.000027 |
| RHOA | -0.6895 | 0.000281 |
| IGFBP2 | 0.0452 | 0.007329 |
| C11orf80 | 1.4984 | 0.002183 |
| ZNF74 | -0.0937 | 0.09058 |
| FOXN2 | -0.9888 | 0.00310 |
| GOLGA8A | 0.658 | 0.000912 |
| RSRP1 | 0.9803 | 0.000262 |
| USP10 | -0.4094 | 0.000207 |
| CLTB | 0.3896 | 0.000154 |
| PIK3R1 | -1.2924 | 0.02166 |
| PABPN1 | 1.5335 | 0.000028 |
| CLCN4 | -0.3669 | 0.019 |
| PARD6B | -1.4978 | 0.001715 |
| TRPA1 | 0.0329 | 0.001898 |
| BAG3 | -0.0174 | 0.0005926 |
| ZNF26 | 0.4511 | 0.0009245 |
| GDPD3 | 0.0381 | 0.002721 |
| SPTBN5 | 1.1286 | 0.0000893 |
| KLHL28 | 2.3647 | 0.0000006 |
| GTPBP8 | 1.042 | 0.001081 |
| TXNDC11 | 2.5667 | 0.0000386 |
| TMEM163 | 0.1489 | 0.003279 |
| Intercept | -12.4231 | / |

p-values are based on t-test
