# Supplementary figures and images for: A genomic-clinicopathologic Nomogram for the preoperative prediction of lymph node metastasis in gastric cancer
Source: BMC Cancer. 2021 Apr 23;21:455. doi: 10.1186/s12885-021-08203-x (PMC8066490; doi:10.1186/s12885-021-08203-x)

A

ROC Curve. Criterion: Youden

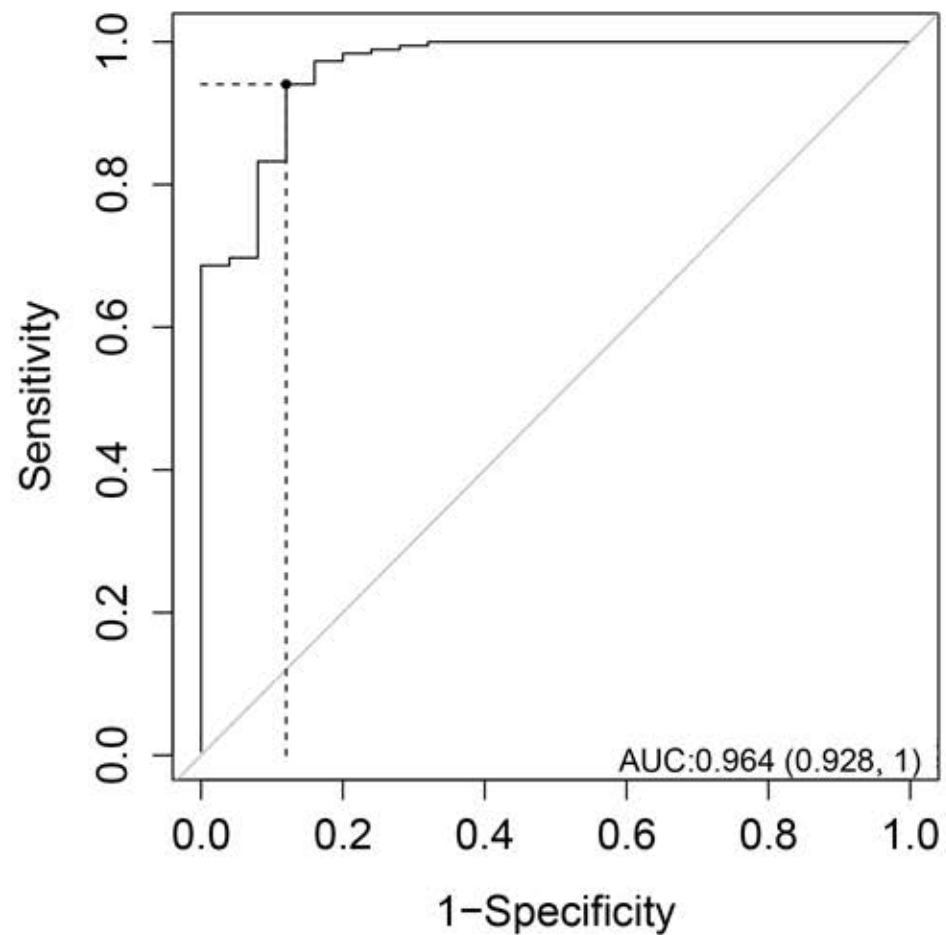

B

PROC Curve. Criterion: Youden

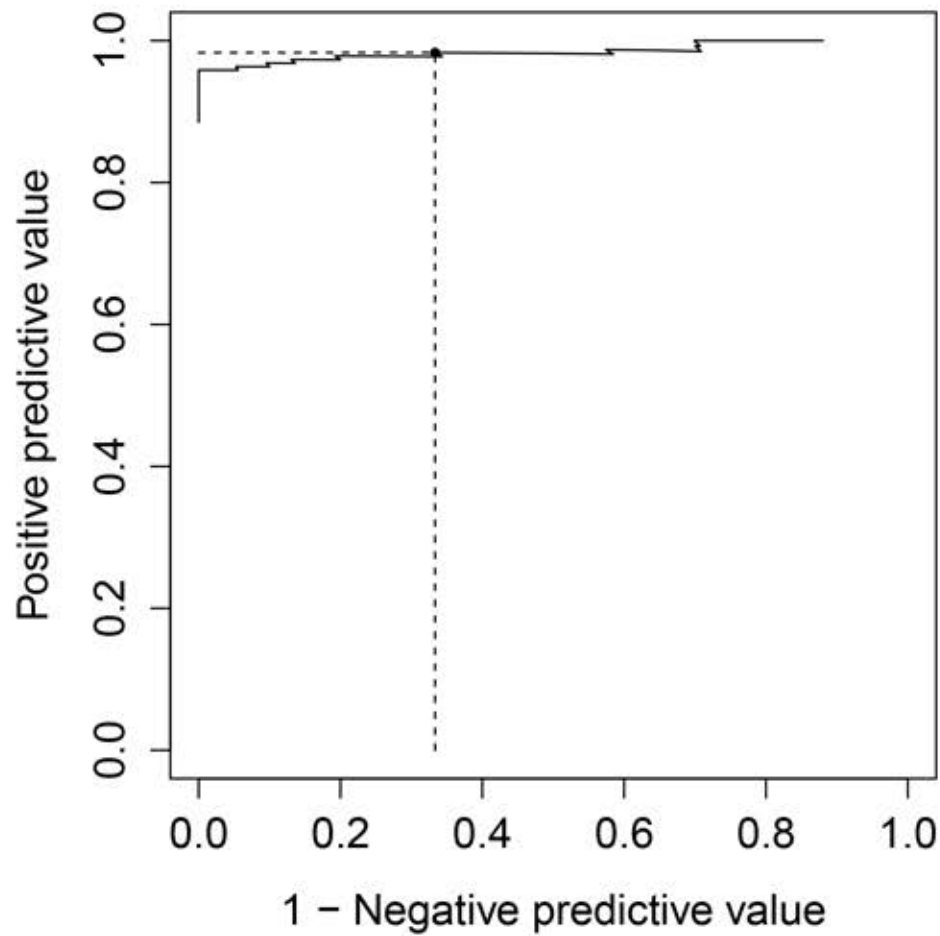

Supplement: Supplementary file 5 — Additional file 5 Figure S1 The optimum cutoff point of risk score. (a) The cutoff value (1.3806) was acquired when the ROC curve reached optimum sensitivity (94.05%) and specificity (88.00%) for predicting LN metastasis. (b) The cutoff value (1.3806) was acquired when Positive Predictive Value (PPV) reached 98%. [file 12885_2021_8203_MOESM5_ESM.pdf]

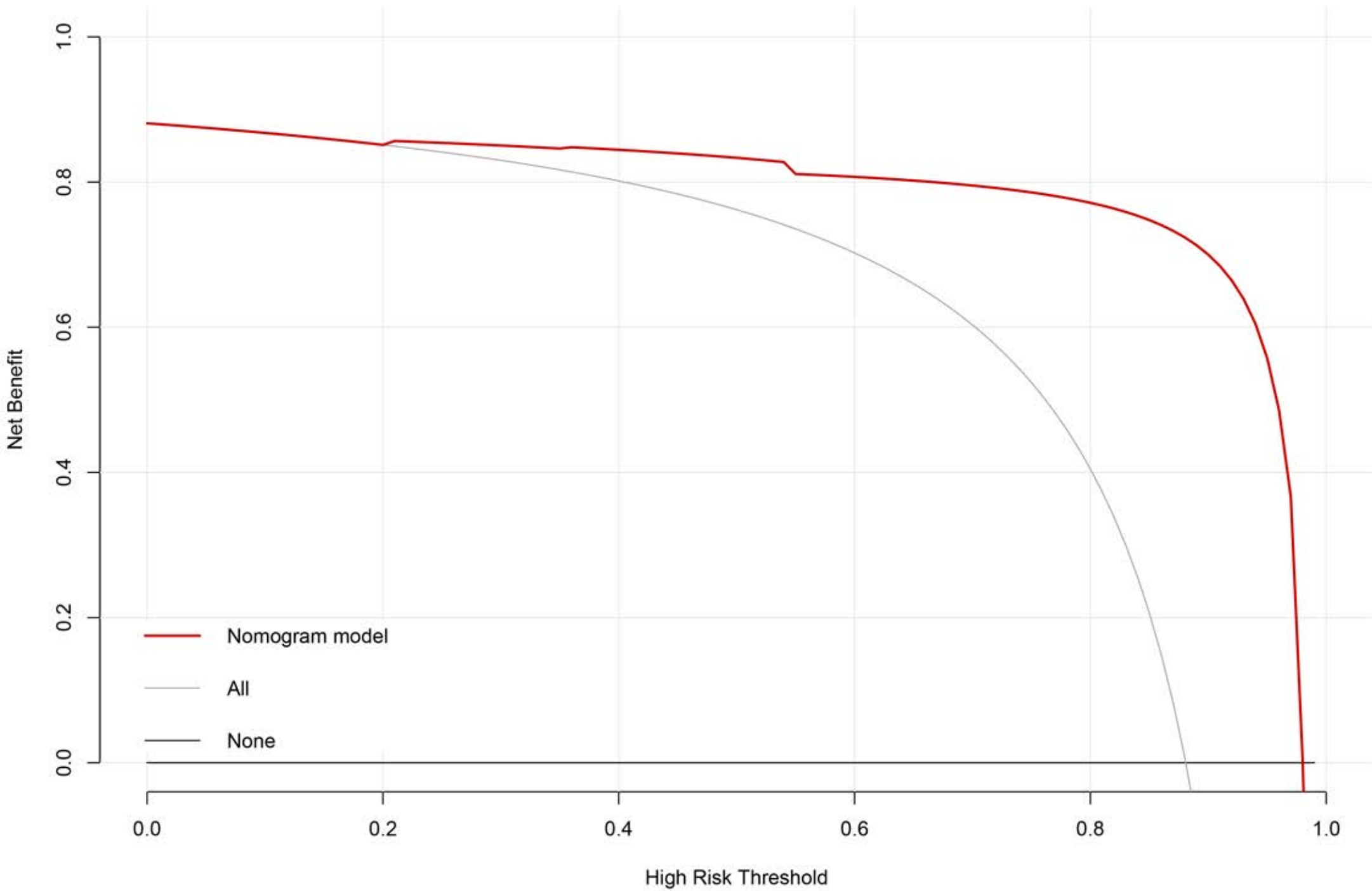

Supplement: Supplementary file 8 — Additional file 8. Figure S2 Decision curve analysis for the genomic signature based nomogram. [file 12885_2021_8203_MOESM8_ESM.pdf]
