# Supplementary material for: A genomic-clinicopathologic Nomogram for the preoperative prediction of lymph node metastasis in gastric cancer
Source: BMC Cancer. 2021 Apr 23;21:455. doi: 10.1186/s12885-021-08203-x (PMC8066490; doi:10.1186/s12885-021-08203-x)
Supplement: Supplementary file 10 — Additional file 10 Fig S3 Network of enriched terms: (a) Colored by the cluster-ID, in which the nodes with similar cluster ID are frequently close to each other. (b) Colored by p-value, in which the terms with more genes tend to have a more remarkable p-value. [file 12885_2021_8203_MOESM10_ESM.pdf]

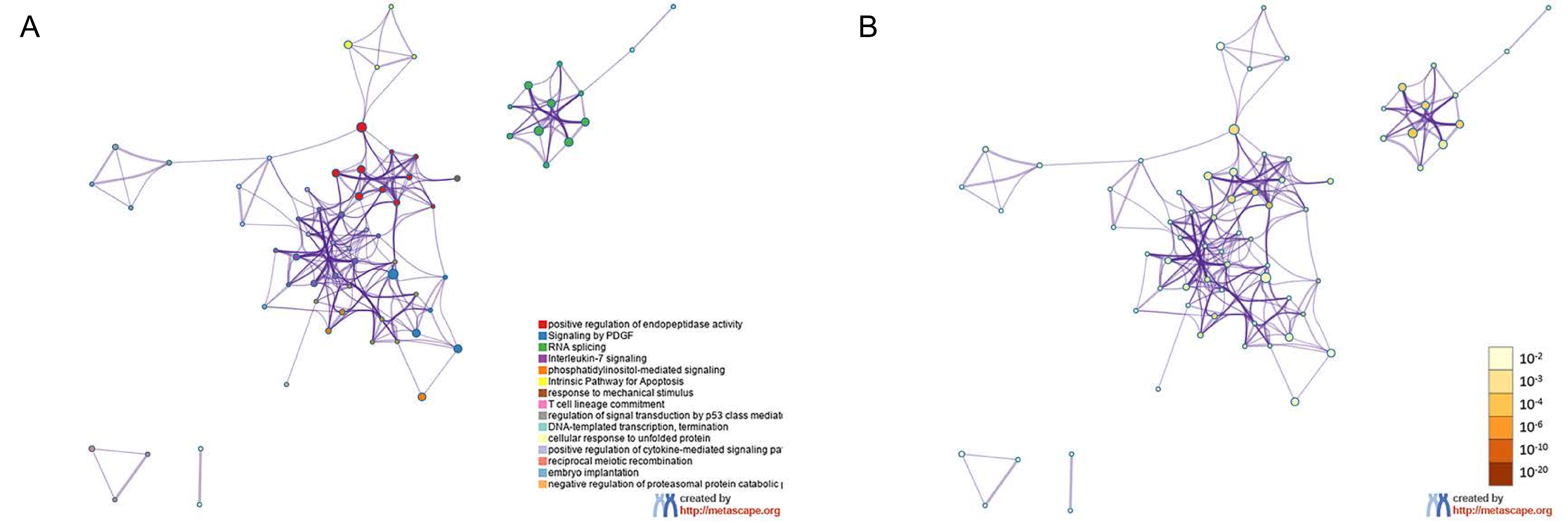
A
B
positive regulation of endopeptidase activity
Signaling by PDGF
RNA splicing
Interleukin-7 signaling
phosphatidylinositol-mediated signaling
Intrinsic Pathway for Apoptosis
response to mechanical stimulus
T cell lineage commitment
regulation of signal transduction by p53 class mediat
DNA-templated transcription, termination
cellular response to unfolded protein
positive regulation of cytokine-mediated signaling pa
reciprocal meiotic recombination
embryo implantation
negative regulation of proteasomal protein catabolic
created by
http://metascape.org
10^{-2}
10^{-3}
10^{-4}
10^{-6}
10^{-10}
10^{-20}
created by
http://metascape.org
